# Alteration of Gene and miRNA Expression in Cervical Intraepithelial Neoplasia and Cervical Cancer

**DOI:** 10.3390/ijms23116054

**Published:** 2022-05-27

**Authors:** Marina Dudea-Simon, Dan Mihu, Laura Ancuta Pop, Razvan Ciortea, Andrei Mihai Malutan, Doru Diculescu, Cristina Alexandra Ciocan, Roxana Maria Cojocneanu, Vasile Simon, Carmen Bucuri, Radu Mocan-Hognogi, Cornelia Braicu, Ioana Berindan-Neagoe

**Affiliations:** 1 2nd Obstetrics and Gynecology Department, “Dominic Stanca” Obstetrics and Gynecology Clinic, “Iuliu Hatieganu” University of Medicine and Pharmacy, 400012 Cluj-Napoca, Romania; dudea.marina@umfcluj.ro (M.D.-S.); dan.mihu@yahoo.com (D.M.); malutan.andrei@gmail.com (A.M.M.); ddiculescu@yahoo.com (D.D.); bucuri.carmen@umfcluj.ro (C.B.); radumocan@yahoo.com (R.M.-H.); 2 Research Center for Functional Genomics, Biomedicine and Translational Medicine, “Iuliu Haţieganu” University of Medicine and Pharmacy, 15 Victor Babeș Street, 400012 Cluj-Napoca, Romania; cristina.ciocan@umfcluj.ro (C.A.C.); cojocneanur@gmail.com (R.M.C.); cornelia.braicu@umfcluj.ro (C.B.); ioana.neagoe@umfcluj.ro (I.B.-N.); 3 Urology Department, “Iuliu Hatieganu” University of Medicine and Pharmacy, 400012 Cluj-Napoca, Romania; vasile.simon@umfcluj.ro

**Keywords:** cervical cancer, cervical intraepithelial neoplasia, long noncoding RNAs, microRNA

## Abstract

**Background**: Cervical cancer is one of the most common malignancies in women in terms of prevalence and mortality. Cervical cancer has some particularities that distinguish it from any other oncologic pathology: first, it is completely preventable by prompt detection of its precursor, cervical intraepithelial neoplasia (CIN); second, the Human Papillomavirus (HPV) infection is a known etiological agent; third, the mean age at diagnosis is much lower than in other oncologic conditions, as a consequence of the sexually-transmitted HPV. **Methods**: We evaluated the expression level of several long noncoding RNAs and a microRNA in samples from 30 patients with CIN, 9 with cervical cancer and 38 normal samples using qRT-PCR technology. **Results**: We observed higher expression levels for *MEG3, DAPK1*, *MLH1* and *MALAT1* in CIN samples than in normal samples, whereas *TIMP3* and *SOX1* had lower expression levels. For cancer samples, *DAPK1*, *MLH1* and *MALAT1* had higher expression, and *MEG3*, *TIMP3* and *SOX1* had lower expression when compared to normal samples. In the case of CIN versus cancer samples, only *MEG3* gene showed a statistically significant difference. The expression of *miR-205-5p* was lower in both CIN and cancer samples compared to normal samples. **Conclusion:** Decreased *MEG3* expression could be considered an alarm signal in the transition from a premalignant cervical lesion to invasive cancer, while altered expression levels of *TIMP3*, *SOX1*, *MLH1*, *MALAT1* and *miR-205-5p* could serve as early biomarkers in the diagnosis of premalignant cervical lesions. Future studies, including a larger number of patients with CIN, will be of particular importance in validating these observations.

## 1. Introduction

Cervical cancer (CC) is one of the most common malignancies in women in terms of prevalence (P) and mortality (Mo), both worldwide (P—4th, Mo—7th in rank) and in Romania (P—3rd, Mo—5th in rank) [1]. CC has some particularities that distinguish it from any other oncologic pathology: first, it is completely preventable by prompt detection of its’ precursor, cervical intraepithelial neoplasia (CIN); second, the human papillomavirus (HPV) infection is a known etiological agent; third, the mean age at diagnosis is much lower than in other oncologic conditions, as a consequence of the sexually-transmitted HPV.

Even though screening programs are available in most countries addressing CIN detection, the incidence and mortality of CC remain high. This is mainly due to risk factors that correlate with the development of CC and the lower number of women participating in the screening program. Certainly, the implementation of CC screening through medical campaigns for low-income women or those without health insurance could reduce the incidence of CC in the future [2].

The main risk factors for CC are related to: persistent infection with high-risk HPV, sexual and reproductive activity pattern (early onset of sexual life, large number of partners, multiparity—all increasing the risk of HPV infection), long-term oral contraception (due to the pro-proliferative effect of estrogen) and smoking (metabolites of cigarette smoke have been found in cervical mucus; modification of cervical mucus is thought to decrease the body’s intrinsic ability to eliminate the HPV infection) [3]. Highly-polymorph cervicovaginal microbiota with decreased Lactobacillus species have recently been shown to promote HPV persistent infection and therefore represent a link in the chain of events that lead to the development of CIN and CC [4,5]. Other pathogens such as *Chlamydia trachomatis*, *Neisseria gonorrheae* and *Herpes Simplex* Virus type 2 have also been proposed, but the correlation with cervical carcinogenesis remains to be investigated [3].

Despite the clinical risk factors mentioned above, it remains unlikely to certify which exposed patients will develop premalignant lesions or CC. Another important aspect regards the features allowing the selection of a target group that would benefit from CIN treatment, considering that not all premalignant cervical lesions evolve towards CC.

The aims of this study are to highlight the mechanisms underlying these transformations, namely altered gene expression, as well as alterations in miRNA expression in patients with CIN and CC compared to patients with a normal cervix. Differences in gene expression between CIN and CC patients as a biomarker of carcinogenesis are also analyzed. The TCGA (The Cancer Genome Atlas) database was analyzed and compared to the information obtained from cases assessed in our institution.

## 2. Results

### 2.1. TCGA Methylation Data Analysis

After sorting the data, we selected *MEG3*, *TIMP3*, *DAPK1*, and *SOX1* as being the most hypermethylated genes specific for the incipient stages of cervical carcinoma, while *MLH1* and *MALAT1* were chosen as the most hypomethylated genes. For this reason, these genes were chosen for further exploration and validation tests.

### 2.2. TCGA Gene and miRNA Expression Analysis

**Figure 1:**
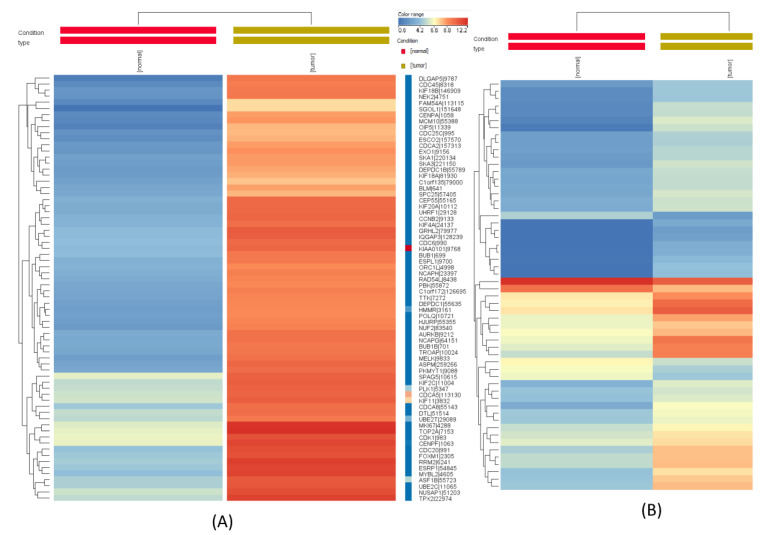
Heatmap for (**A**) gene expression alterations in tumor vs. peritumoral tissue; (**B**) miRNA expression alterations in tumor vs. peritumoral tissue (the red frame shows *miR-205-5p* as being overexpressed).

When comparing gene expression in tumor samples to peritumoral normal tissue, the analysis revealed a high number of differentially expressed genes (2178 downregulated and 1810 upregulated) (Figure 1A), while the same analysis for microRNAs returned a much lower number of molecules that passed the cut-off criteria. Thus, we obtained a total number of 207 differentially expressed miRNAs, of which 24 were downregulated, and 183 upregulated (Figure 1B).

The most upregulated microRNA was *hsa-miR-205-5p*, which was also observed as being the second most upregulated miRNA in the differential expression analysis of samples from HPV positive patients compared to samples from HPV negative patients (the most upregulated being *hsa-miR-944*). For this reason, *miR-205-5p* was chosen for further exploration and validation tests.

**Figure 2:**
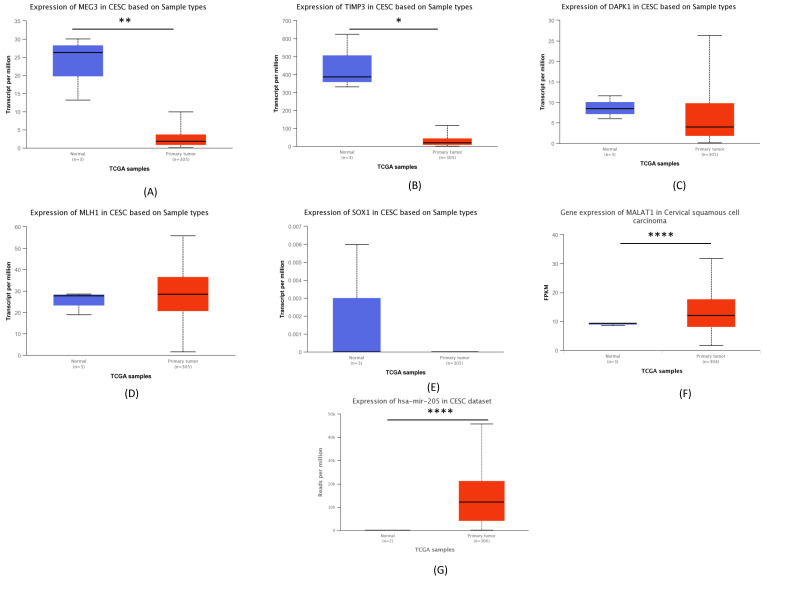
Expression levels of (**A**) *MEG3,* (**B**) *TIMP3*, (**C**) *DAPK1*, (**D**) *MLH1*, (**E**) *SOX1*, (**F**) *MALAT1* and (**G**) *miR-205-5p* in the TCGA dataset. (* *p* ≤0.05, ** *p* ≤0.01, **** *p* ≤0.0001).

When analyzing the expression level of the selected genes from the TCGA methylation analysis in TCGA dataset, we observed a statistically significant alteration in the expression level of *MEG3*, *TIMP3* and *MALAT1*. In addition, we noted that in tumor samples the expression level of *MEG3*, *TIMP*, *DAPK1* and *SOX1* was lower, while the expression of *MLH1* and *MALAT1* was higher in comparison to the normal samples. The expression level of *mir-205-5p* was significantly higher in cancer samples compared to normal samples (Figure 2).

**Figure 3:**
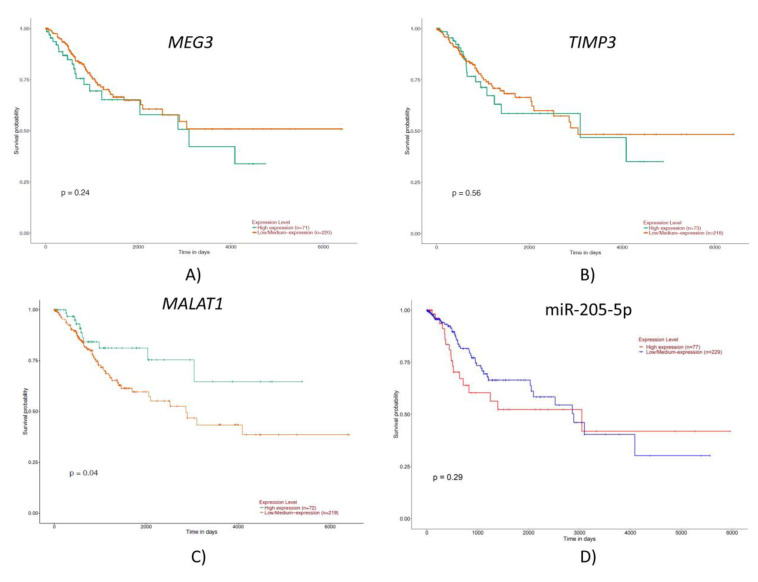
Survival curves for (**A**) *MEG3*, (**B**) *TIMP3*, (**C**) *MALAT1* and (**D**) *miR-205-5p*.

When analyzing the survival curves, the following tendency was noted: higher expression of *MEG3*, *TIMP3* and *MALAT1* was correlated to a lower survival rate, while lower expression of *miR-205-5p* was correlated to a lower survival rate, even though the data were not statistically significant, except for *MALAT1* (Figure 3).

### 2.3. Gene Expression Analysis

**Figure 4:**
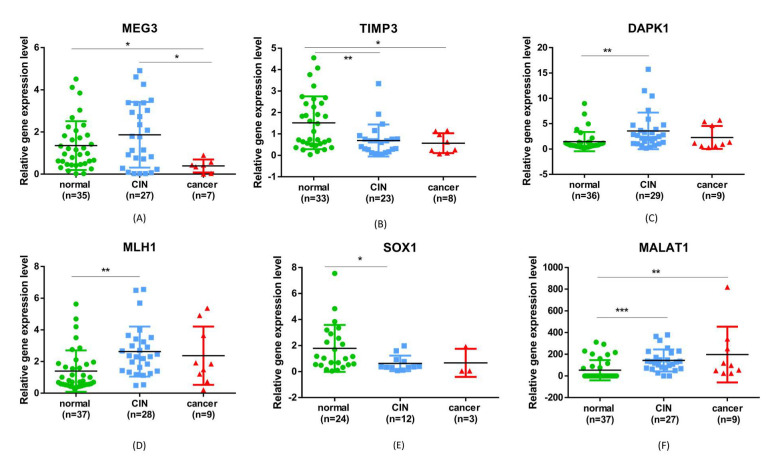
Expression levels of (**A**) *MEG3*, (**B**) *TIMP3*, (**C**) *DAPK1*, (**D**) *MLH1*, (**E**) *SOX1* and (**F**) *MALAT1* for normal, cervical intraepithelial neoplasia (CIN) and cancer samples. (* *p* ≤ 0.05, ** *p* ≤ 0.01, *** *p* ≤ 0.0001).

In the analysis of the relative expression level of the tested gene, we obtained statistically significant levels for *TIMP3*, *DAPK1*, *MLH1*, *SOX1* and *MALAT1* when comparing normal to CIN samples. In the case of normal vs. cancer samples, significant differences were observed for the expression of *MEG3*, *TIMP3* and *MALAT1*. The expression level of the selected gene was also compared between CIN and cancer samples, revealing significant differences for the *MEG3* gene (Figure 4). For CIN samples, the expression level of *MEG3*, *DAPK1*, *MLH1* and *MALAT1* was higher than in normal samples, while *TIMP3* and *SOX1* had lower expression. For cancer samples, the expression level was higher than in normal samples for *DAPK1*, *MLH1* and *MALAT1*, and lower for *MEG3*, *TIMP3* and *SOX1*.

**Figure 5:**
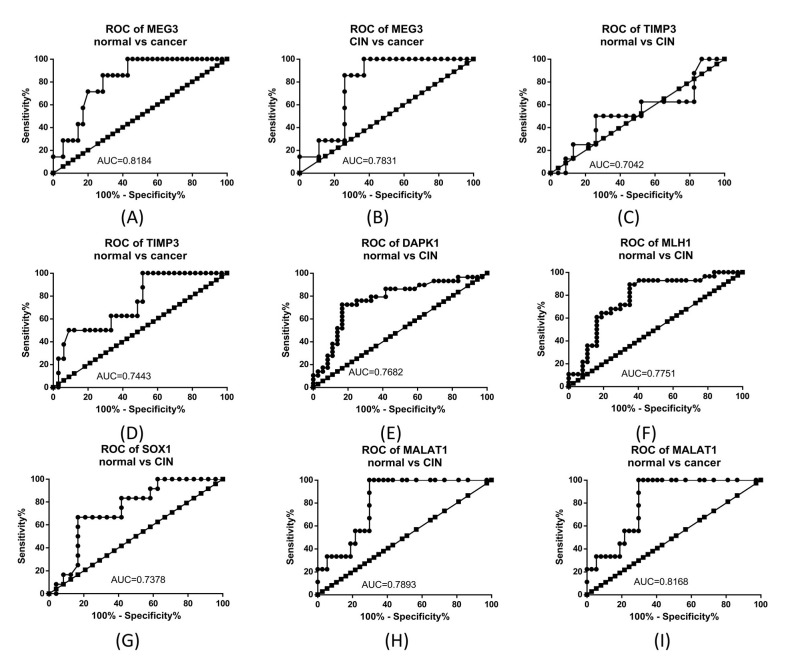
Receiver operating characteristic (ROC) curves for (**A**) *MEG3* normal vs. cancer; (**B**) *MEG3* CIN vs. cancer; (**C**) *TIMP3* normal vs. CIN; (**D**) *TIMP3* normal vs. cancer; (**E**) *DAPK1* normal vs. CIN; (**F**) *MLH1* normal vs. CIN; (**G**) *SOX1* normal vs. CIN; (**H**) *MALAT1* normal vs. CIN; and (**I**) *MALAT1* normal vs. cancer.

When analyzing the Receiver operating characteristic (ROC) curves for these genes we noted that, for the comparison of normal to cancer samples, *MEG3* and *MALAT1* had the highest Area Under the Curve (AUC), while when comparing normal vs. CIN, *MLH1* and *DAPK1* presented the highest AUC. Figure 5 presents the ROC curves for the comparison between normal, CIN, and cervical cancer in the cases where the gene expression difference was statistically significant.

### 2.4. miR-205-5p Expression

**Figure 6:**
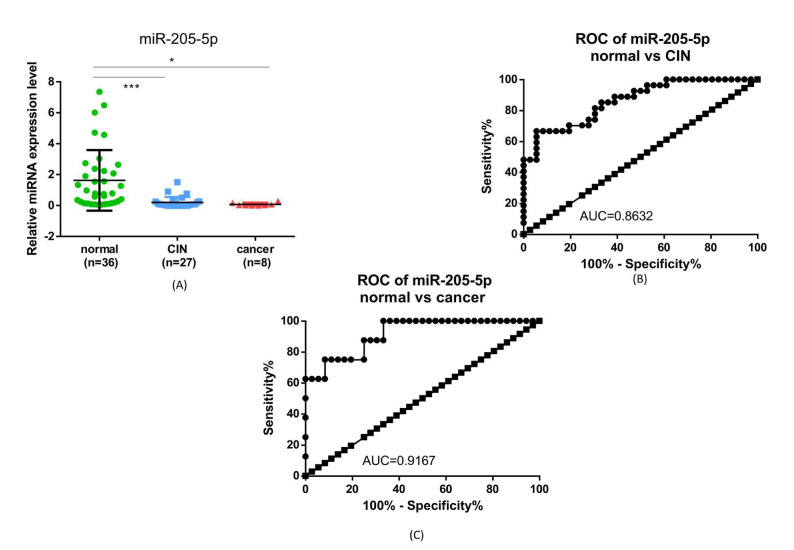
(**A**) Expression of miR-205-5p in selected samples; (**B**) ROC curve for miR-205-5p in normal vs. CIN samples; (**C**) ROC curves for miR-205-5p in normal vs. cancer samples. (* *p* ≤ 0.05, *** *p* ≤ 0.0001).

Regarding the expression level of *miR-205-5p*, it is noted that the expression is significantly lower in the CIN and cancer groups, compared to normal samples. In addition, the AUC of the ROC curve for this biomarker was 0.78632 for CIN and 0.9167 for cancer (Figure 6).

### 2.5. ROC Curves for the Combination of Candidate Targets

**Figure 7:**
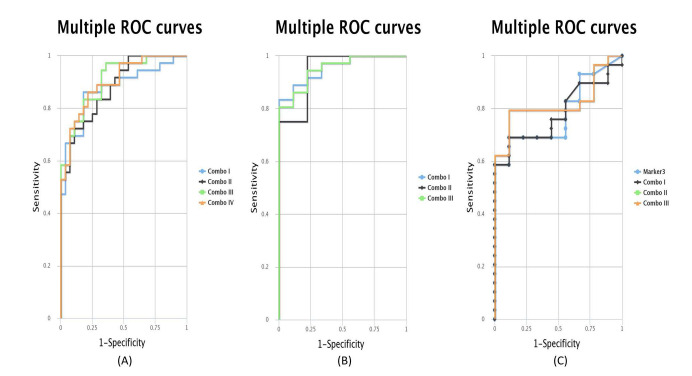
ROC curves for combinations of targets for (**A**) normal vs. CIN samples, (**B**) normal vs. cancer samples, and (**C**) CIN vs. cancer samples.

Seeing that the individual candidate genes and miRNA are not great predictors, we evaluate also the combination of candidate targets. Using the CombiROC software, we were able to evaluate each combination of targets and select the ones with the best AUC. As can be seen in Figure 7 for normal vs. CIN samples, the best combinations were between *MEG3*, *MLH1*, *TIMP3* and *miR-205-5p* and *MEG3*, *TIMP3* and *miR-205-5p* for CIN vs. cancer and CIN vs. cancer samples.

Table 1 presents the exact combinations that have the highest AUC and the AUC values for each combination of samples.

### 2.6. Correlation of the Expression Data

Table 2 presents the statistical correlation between the expressions of the tested targets. The values in red represent correlations that are statistically significant. A direct significant correlation was observed between the expression of *MEG3* and *DAPK1*, *MLH1*, *MALAT1* and *miR-205-5p*, *TIMP3* and *DAPK1*, *DAPK1* with *MLH1*, *MALAT1* and *miR-205-5p*, *MLH1* and *MALAT* and *miR-205-5p*.

## 3. Discussion

The peculiarity of CC is that it is preceded by a premalignant lesion identifiable by pathological examination. This is of particular importance, given the significant prevalence of CC in younger age groups compared to other oncological pathologies [6]. While several studies focused on identifying core factors in the evolution of CC, the genesis of premalignant cervical lesions is not fully unraveled [7,8,9,10]. CIN represents a condition often identified in young patients of reproductive age. In the absence of certain predictors for the evolution of CIN, the necessary treatments may have an impact on the patient’s reproductive future.

Despite some clinical and virological risk factors identified a few decades ago, the molecular mechanism by which a certain group of women exposed to them develop CIN or CC is not fully elucidated.

The present study highlighted the most hypo- and hypermethylated genes specific to the early stages of cervical carcinoma, and subsequently the most over- or underexpressed genes and miRNAs in patients with premalignant or malignant lesions of the cervix. For this purpose, the data of 304 patients with CC were analyzed, whose information was available by accessing the TCGA online database. To validate this information, as well as to compare the changes identified in CC with those present in CIN, 77 patients admitted to our institution were enrolled in the study, for which samples of affected and unaffected cervical tissue were processed and analyzed, confirmed by histopathological examination.

Currently, persistent HPV infection is considered the main risk factor responsible for the onset of CIN and subsequently for the development of CC. The inefficient immune response has a substantial role in the HPV persistence, as shown by several papers reporting altered gene expression with increase in pro-proliferative gene levels, such as CDKN2A, and low expression of genes related to a good immune response (NCAM1), leading to inhibition of pro-inflammatory molecular cascade in the surrounding stromal environment in CIN and early CC stages [11,12,13].

However, despite these considerations, the individual response to HPV and the evolutionary nature of CIN are still unpredictable: CIN 1—57% regression, 32% persistence, 11% CIS (carcinoma in situ), 1% CC; CIN 2—43% regression, 35% persistence, 22% CIS, 5% CC; CIN 3—32% regression, <56% persistence, >12% invasion [14,15].

Therefore, given the seemingly unpredictable evolutionary nature of CIN, the mechanisms underlying carcinogenesis are expected to be much more complex. The identification of specific changes in gene expression and miRNA could fundamentally contribute to the understanding of these mechanisms and to the future development of targeted therapeutic strategies, including the absence of the need for active intervention in selected cases in patients diagnosed with CIN. This is of particular importance given the significant potential for adverse obstetric events in a pregnant patient with a history of cervical surgery.

Another aspect of this research was to identify over- and underexpressed genes, as well as miRNAs with altered expression level, in CIN patients compared to patients with a normal cervix: persistent HPV infection may or may not generate CIN. A deeper understanding of these early changes will allow targeted therapeutic approaches towards the development of CIN.

For this purpose, the use of the TCGA database allowed the selection of genes and miRNAs whose expression was tested and compared between patients with normal, premalignant and malignant cervix. Certain patterns were highlighted, indicated by TCGA data and confirmed by validation tests.

Thus, the reduction of the expression level of *TIMP3* and *SOX1* compared to normal tissue was present both in the TCGA analysis and in the patients enrolled in the validation study, in the CC and CIN group. The *TIMP3* gene (*TIMP Metallopeptidase Inhibitor 3*) encodes inhibitory proteins of matrix metalloproteinases. Decreased expression of this gene leads, implicitly, to increased extracellular matrix degradation activity, promoting the development of cyto-architectural abnormalities, which is a known factor in promoting carcinogenesis [16]. Alteration of the stromal microenvironment in CIN and CC has been the research topic of several papers [12,17,18]. Furthermore, a clinical study assessing the stromal stiffness, underlying the epithelium, in patients with normal, premalignant and malignant cervix objectified by elastography the increase in stromal stiffness in patients with CIN and cervical cancer, compared to normal [19]. A possible underlying explanation was the modification of the stromal microenvironment since the stage of premalignant lesions, with the alteration of the extracellular matrix. Indeed, this observation is supported by our results, by objectifying the low level of *TIMP3* expression.

The *SOX1* gene (*SRY-Box Transcription Factor 1*) encodes a transcription factor involved in embryonic development and cell fate. Hypermethylation of this gene, resulting in decreased expression, has been identified as an independent predictor in relation to HPV-HR infection for CIN development, being therefore considered a promising biomarker for high-grade premalignant lesions [20]. Our results support this statement, the reduced expression of this gene being confirmed by validation tests in CC and CIN groups.

Similarly, hypomethylation causing *MLH1* and *MALAT1* overexpression was detected in patients with CC by analyzing the TCGA data set and validated for both the CC group and the CIN group, compared to patients with a normal cervix. Although *MLH1* is a tumor suppressor gene involved in the repair of DNA defects, mutations in this gene have been associated with DNA binding defects and the development of gene instabilities found in conditions such as Lynch syndrome, including colorectal, endometrial, cervical, esophageal, skin and breast cancer [21]. *MALAT1* (*Metastasis Associated Lung Adenocarcinoma Transcript 1*) encodes a precursor transcript, which regulates the transcription of numerous genes, including some involved in cell migration, cell cycle regulation, and the process of metastasis. Its overexpression, found in multiple cancers, has been associated with cell proliferation and the development of metastases [22].

An important finding of this study is represented by the changes in the expression level of the *MEG3* gene (*Maternally Expressed 3*). Transcripts of this gene are involved in inhibiting cell proliferation, tumor growth, and metastasis [23,24]. According to our data, the expression level of *MEG3* does not differ between normal and CIN, but is significantly lower in patients with cervical cancer, both compared to the group of patients with normal cervical cancer and that of patients with CIN. Therefore, a low level of expression of this gene could be an alarm signal for the transition from CIN to CC. Our affirmation is supported by the study conducted by He et al. (2017), which suggests the use of MEG3 levels in the diagnosis and prognosis of malignant conditions [25].

The analysis of the expression of the DAPK1 gene (*Death-Associated Protein Kinase 1*), a tumor suppressor gene, mediator of programmed cell death induced by gamma-interferon, provided contradictory results. According to the analysis of the TCGA data set, hypermethylation of its promoter was found, translated into a low level of gene expression. Indeed, its involvement in the pathogenesis of CIN and CC is supported by previous publications [26,27]. When analyzing its level in the validation samples, our results indicate a slight increase in DAPK1 expression, more expressed in the CIN group than in the CC group, compared to the group of patients with a normal cervix. Further studies, enrolling more patients in our geographical area, could clarify this aspect.

Unlike the change in MEG3 levels at the transition from CIN to an invasive form, the *TIMP3*, *SOX1*, *MLH1*, *MALAT1* genes are expressed at a different level than normal in both CC and CIN. These genes could be used as early biomarkers of premalignant cervical lesions.

Regarding the change in miRNA expression level in CIN and CC, the present study identified *miR-205-5p* as being significantly lower in the samples of patients with CIN and CC compared to the normal samples. Its value as a diagnostic test was assessed by plotting the ROC curves, with very good results, with an AUC of 0.78 for CIN and 0.91 for CC, respectively. From the TCGA database, we also identified other miRNAs that were correlated to CC, as shown in Figure 1B. However, we only selected *miR-205-5p* to validate our cohort of patients because of its’ marked differential expression, lower in CC than in normal tissue and because it is correlated to the tested genes. Multiple other miRNAs have been proposed as biomarkers, of which *miR-21* overexpression detected in cervical tissue and patient serum, *miR-20a*, *miR-9*, *miRNA-10a*, and reduced expression level for *miR-19a*, *miR-22*, *miR-27a*, *miR-100* [28,29,30,31,32,33]. Laengsri et al. systematized this information in a comprehensive review, classifying changes in miRNA expression according to the geographical region of the origin of the patients enrolled in the study [34].

## 4. Materials and Methods

### 4.1. Selection of Study Group

For the analysis of TCGA data, the information of 304 patients with CC was used; all available clinical data of these patients is presented in Table 3. Regarding the patients used for data validation, 9 CC patients were enrolled in the study, 30 with CIN and 38 patients with a normal cervix, hospitalized at the *“Dominic Stanca” Obstetrics-Gynecology Clinic, Cluj-Napoca, Romania*. For these patients, the diagnosis was confirmed by pathological examination obtained as a result of a cervical biopsy or other surgery on the cervix—conization or hysterectomy (Table 1). The project and protocol of this prospective study were approved by the Ethics Committee of the *University of Medicine and Pharmacy “Iuliu Hațieganu”, Cluj-Napoca, Romania* (96/63/08 March 2017). After explaining the nature of the study and the procedures involved, verbal and written informed consent was obtained from all participants.

### 4.2. TCGA Methylation Data Analysis

The methylation data for cervical squamous cell carcinoma and endocervical adenocarcinoma (CESC), together with clinical information, and expression data for microRNAs and messenger RNA, come from the TCGA (The Cancer Genome Atlas) project, and was downloaded from the Firebrowse website (firebrowse.org (accessed on 4 March 2021)) hosted by the Broad Institute of MIT and Harvard. The methylation data was in the form of a matrix that contains information for 20,108 genes and 312 biological samples, some of which are tumor tissues, and some metastatic, or peritumoral normal tissue. For the methylation study, we took into consideration the beta values for the tumor tissues, which were averaged, and the genes were sorted in descending order according to these values. The methylation beta values and their average are subunitary, with the values closer to zero representing hypomethylation, and the ones that grow towards one representing hypermethylated genes.

### 4.3. TCGA Gene and miRNA Expression Analysis

We also used the UALCAN database for TCGA cervical cancer analysis [35]. The cervical cancer database consists of 305 tumor samples and 3 normal samples. In this analysis we evaluated the expression level and survival profile of *MEG3*, *TIMP3*, *DAPK1*, *MLH1*, *SOX1*, *MALAT1* and *miR-205-5p*.

### 4.4. RNA/DNA Extraction

For the RNA and DNA extraction from the tissue samples obtained from the participants included in the study from our institution, we used the TriReagent (Invitrogen, Waltham, MA, USA) classical method, as described by the manufacturer. The RNA was extracted from normal and tumor tissue after mechanic homogenization in TriReagent (Ambion, Austin, TX, USA).

In short, for RNA extraction we used 160 µL of chloroform to the 800 µL of TriReagent, then the samples were vortexed vigorously and incubated at room temperature for 15 min. After incubation, the samples were centrifuged for 15 min at 14,000 rpm and 4 °C. After centrifugation, three layers were noted: aqueous layer (containing RNA), organic layer (containing DNA), and phenol layer (containing proteins). The aqueous layer was transferred to a clean tube, and we added 500 µL of 2-propanol to precipitate the RNA. The tube was inversed in order to mix the solutions and then left at room temperature to incubate for 10 min. Afterwards, the samples were centrifuged for 10 min at 14,000 rpm and 4 °C. The supernatant was removed and the RNA pellet was washed with 500 µL of 75% ethanol, mixed by vortexing and then centrifuged for 5 min at 14,000 rpm and 4 °C. After centrifugation, all the ethanol was removed and the pellet was left to air dry for 10 min. After the pellet was dried, we added 20 ng of nuclease free water to dissolve the pellet. The RNA was quantified using Nanodrop (ThermoFisher Scientific, Waltham, MA, USA). The quantity of RNA was approximately 7418 ng/µL.

### 4.5. Gene Expression Analysis

The extracted RNA samples (75 samples: 9 cervical cancer, 29 CIN and 37 normal tissue) were used for the evaluation of the expression levels of the following genes: *MEG3*, *TIMP3*, *DAPK1*, *MLH1* and *SOX1*, and the long non-coding *MALAT1*. First, the RNA was subjected to a DNase treatment using Turbo DNase (Ambion Austin, TX, USA). We started with 1500 ng of RNA in 8.5 µL of water and added 1 µL of Turbo DNase Buffer, 0.5 µL of Turbo DNase and 0.25 µL of RNase inhibitor. This mixture was incubated at 37 °C for 30 min. Afterwards, 2 µL of DNase inhibitor was added and the mixture was incubated at room temperature for 5 min. The samples were then centrifuged at 14,000 rpm for 2 min and the supernatant was transferred to a new tub and used in the cDNA synthesis reaction. For cDNA synthesis, the High-Capacity cDNA Reverse Transcription Kit (Applied Biosystems, Waltham, MA, USA) was used. To the supernatant obtained in the DNase treatment reaction we added 2 µL of 10XRT buffer, 0.8 µL of 25X dNTP, 2 µL of 10X Random Primer, 1 µL of Multiscribe Reverse Transcriptase, 0.25 µL RNase inhibitor and 3.95 µL of nuclease free water. This mixture was incubated: 10 min at 25 °C, 120 min at 37 °C, 5 min at 85 °C, and held at 4 °C. After cDNA synthesis, the obtained cDNA was diluted 1:5 with nuclease free water. For gene expression analysis, we used the ready-to-use SYBR Select Master Mix (Applied Biosystems Waltham, MA, USA) and 2.5 µL diluted cDNA. Over the cDNA, we added 10 µL of Sybr Select Master Mix, 0.1 µL of primer 100 µM or 0.2 µL of primers 50 µM and 8.8/8.6 µL of nuclease free water. This mixture was split into two wells of the PCR plate, 10 µL in each well. The PCR program used in the ViiA 7 (Applied Biosystems, Waltham, MA, USA) instrument was as follows: 1 cycle—2 min at 50 °C, 1 cycle—2 min at 95 °C and 40 cycles at 95 °C–15 s and 60 °C–30 s. The obtained C_T_ values were analyzed using the ΔΔC_T_ method and the obtained results were imported into GraphPad Prism software (GraphPad, San Diego, CA, USA) for further analysis.

### 4.6. miR-205-5p Expression Analysis

The total RNA extracted (77 samples: 9 cervical cancer, 30 CIN and 38 normal tissue) was diluted to 50 ng/µL and used together with the TaqMan microRNA transcription kit (Applied Biosystems, Waltham, MA, USA) and the TaqMan microRNA primer assay for *miR-205-5p*, *U6* and *RNU48* (ThermoFisher Scientific Waltham, MA USA). The microRNAs *U6* and *RNU48* were used as housekeeping microRNAs. Therefore, 1 µL of total RNA was mixed with 0.75 µL of 10X RT Buffer, 0.1 µL of RNase inhibitor, 0.075 µL dNTP, 0.1825 µL of each of the 20X miRNA RT primers, 4.52 µL of nuclease free water and 0.5 µL of MultiScribed RT enzyme. The mixture was incubated at: 16 °C for 30 min, 42 °C for 30 min, 85 °C for 5 min and held at 4 °C. The cDNA obtained was diluted three times with nuclease free water and then used in the real time PCR reaction. We prepared a mixture of 5.03 µL of ready to use TaqMan Fast Advance Master Mix (Applied Biosystems, Waltham, MA, USA) and 0.47 µL of TaqMan microRNA primer, and added 5.2 µL of cDNA for each of the miRNA analyzed. From this mixture, we added 5 µL to two wells of the PCR plate. The PCR program used in the Viia 7 (Applied Biosystems, Waltham, MA, USA) instrument was as follows: 1 cycle—2 min at 50 °C, 1 cycle—20 s at 95 °C and 40 cycles at 95 °C–1 s and 60 °C–20 s in the FastMode. The obtained C_T_ values were analyzed using the ΔΔC_T_ method and the obtained results were imported in GraphPad Prism software (GraphPad, San Diego, CA, USA) for further analysis.

### 4.7. ROC Curve Analysis Using CombiROC Sofrware

For the analysis of ROC curves of combination targets, we used the CombiROC software [36] for the selected groups of patients, using test signal cutoff 4 and minimum feature 1.

### 4.8. Statistical Analysis

For the statistical analysis of the expression data and correlations of the expression data, the GraphPad Prism version 6.0 for Windows (GraphPad, San Diego, CA, USA) software was used.

## 5. Conclusions

The results of this study support the presence of changes in the expression level of genes and miRNA, as part of the pathogenesis of CIN and CC. Decreased *MEG3* expression could be considered an alarm signal in the transition from a premalignant cervical lesion to invasive cancer, while altered expression levels of *TIMP3*, *SOX1*, *MLH1*, *MALAT1* and *miR-205-5p* could serve as early biomarkers in the diagnosis of premalignant cervical lesions. Future studies, including a larger number of patients with CIN, will be of particular importance in validating these observations.

## Figures and Tables

**Table 1:** The Area Under the Curve (AUC) values for the best predictive combinations.

Normal vs. CIN			Normal vs. Cancer			CIN vs. Cancer		
Combination	Candidates	AUC	Combination	Candidates	AUC	Combination	Candidates	AUC
Combo I	*MEG3* + *MLH1* + *miR-205-5p*	0.876	Combo I	*MEG3* + *miR-205-5p*	0.954	Combo I	*MEG3* + *TIMP3*	0.782
Combo II	*MEG3* + *TIMP3* + *miR-205-5p*	0.881	Combo II	*TIMP3* + *miR-205-5p*	0.944	Combo II	*MEG3* + *miR-205-5p*	0.82
Combo III	*MLH1* + *TIMP3* + *miR-205-5p*	0.907	Combo III	*MEG3* + *TIMP3* + *miR-205-5p*	0.951	Combo III	*MEG3* + *TIMP3* + *miR-205-5p*	0.82
Combo IV	*MEG3* + *MLH1* + *TIMP3* + *miR-205-5p*	0.9						

**Table 2:** Statistical correlation between the expressions of the tested targets.

*p* Value	*MEG3*	*TIMP3*	*DAPK1*	*MLH1*	*SOX1*	*MALAT1*	*miR-205-5p*
*MEG3*		0.9832	0.0005	1.74 × 10^−14^	0.8237	9.11 × 10^−5^	2.89 × 10^−5^
*TIMP3*	0.9832		0.0012	0.8836	0.7740	0.0981	0.9408
*DAPK1*	0.0005	0.0012		5.49 × 10^−10^	0.4147	5.20 × 10^−14^	0.0033
*MLH1*	1.74 × 10^−14^	0.8836	5.49 × 10^−10^		0.6617	4.36 × 10^−19^	0.0199
*SOX1*	0.8237	0.7740	0.4147	0.6617		0.6888	0.7545
*MALAT1*	9.11 × 10^−5^	0.0981	5.20 × 10^−14^	4.36 × 10^−19^	0.6888		0.3439
*miR-205-5p*	2.89 × 10^−5^	0.9408	0.0033	0.0199	0.7545	0.3439	

The values displayed in red indicate a statistically significant correlation.

**Table 3:** Clinical data of patients used in the analysis of The Cancer Genome Atlas TCGA data, as well as in the analysis of validation tests.

Demographics		CESC (*n* = 304)	Cervical Cancer (9)	CIN (30)	Normal (38)
Age	Median, Range ♀	46, 20–88	43, 29–64	39, 22–63	44, 28–65
HPV Status	Positive	281	9	29	30
	Negative	22	0	1	8
	Indeterminate	1	0		
Pathologic TNM	T1	140	5		
	T2	71			
	T3	20			
	T4	10			
	Tis	1			
	Tx	17			
	T unknown	45	4		
	N0	133	5		
	N1	60			
	Nx	66			
	N unknown	45	4		
	M0	116	5		
	M1	10			
	Mx	128			
	M unknown	50	4		
Clinical stage	I	162	1		
	II	69	2		
	III	45	1		
	IV	21			
	Unknown	7	5		
Birth control pill use	Current user	15	1	4	4
	Former user	53	3	12	15
	Never used	89	5	14	19
	NA	147			
Histological type	Adenosquamous	5			
	Cervical squamous cell carcinoma	252	9		
	Endocervical adenocarcinoma of the usual type	6			
	Endocervical type of adenocarcinoma	21			
	Endometroid adenocarcinoma of endocervix	3			
	Mucinous adenocarcinoma of endocervical type	17			
Tobacco smoking history	Lifelong non-smoker	144	5	17	24
	Current smoker	64	4	13	14
	Reformed smoker > 15 years	9			
	Reformed smoker ≤ 15 years	40			
	Reformed smoker duration unknown	4			
	NA	43			

CESC—cervical squamous cell carcinoma and endocervical adenocarcinoma; CIN—cervical intraepithelial neoplasia; HPV—human papillomavirus, TNM Tumor, Nodes, Metastases, NA—not available.

## Data Availability

Not applicable.
